# Digestibility of crude nutrients and minerals in C57Bl/6J and CD1 mice fed a pelleted lab rodent diet

**DOI:** 10.1038/s41598-024-52271-5

**Published:** 2024-01-20

**Authors:** Linda F. Böswald, Dana Matzek, Bastian Popper

**Affiliations:** Core Facility Animal Models, Biomedical Center, Ludwig-Maximilians-Universität München, Munich, Germany

**Keywords:** Physiology, Animal physiology

## Abstract

In laboratory animals, there is a scarcity of digestibility data under non-experimental conditions. Such data is important as basis to generate nutrient requirements, which contributes to the refinement of husbandry conditions. Digestibility trials can also help to identify patterns of absorption and potential factors that influence the digestibility. Thus, a digestibility trial with a pelleted diet used as standard feed in laboratory mice was conducted. To identify potential differences between genetic lines, inbred C57Bl/6 J and outbred CD1 mice (n = 18 each, male, 8 weeks-old, housed in groups of three) were used. For seven days, the feed intake was recorded and the total faeces per cage collected. Energy, crude nutrient and mineral content of diet and faecal samples were analyzed to calculate the apparent digestibility (aD). Apparent dry matter and energy digestibility did not differ between both lines investigated. The C57Bl/6 J mice had significantly higher aD of magnesium and potassium and a trend towards a lower aD of sodium than the mice of the CD1 outbred stock. Lucas-tests were performed to calculate the mean true digestibility of the nutrients and revealed a uniformity of the linear regression over data from both common laboratory mouse lines. The mean true digestibility of crude nutrients was > 90%, except for fibre, that of the minerals ranged between 66 and 97%.

## Introduction

In laboratory animal science, the concept of the 3Rs—Replacement, Reduction, Refinement—is an important framework^1^. Refinement comprises optimizing husbandry, environment and experimental procedures^2–4^. The prerequisite to ensure animal welfare and to avoid unnecessary harm is the understanding of the basic requirements and physiology of a species under “normal”, maintenance conditions^4^. This is also important as basis of all experimental planning. Another “R” has been added to the list, namely the Responsibility of the research community towards the laboratory animals^5^. The responsibility extends to animal welfare and health, including a nutrition that meets the requirements of the respective species.

The current recommendations for nutrient supply for laboratory animal species have been extrapolated from empirical data and singular studies summarized by the National Research Council (NRC) in 1995^6^. The authors state that “too few controlled studies have been conducted […] to identify the nutrient requirements” of mice, and part of the recommendations are based on values for rats^6^. In addition, the studies that contributed to the recommendations were conducted in the second half of the twentieth century. Since then, laboratory animal husbandry conditions and diet manufacturing have changed, which can influence the nutritional demands. Thus, it is important to find out more about the basic digestive physiology of laboratory mice under today’s standard conditions. Determining the energy digestibility is the basis to reach the first stage of a species-specific energy evaluation, existing for dogs, cats, pigs, horses and ruminants^7,8^, which helps to predict the energy that can be utilized by an animal from the feed.

Diet and nutrient digestibility is influenced by many species-specific and dietary factors^7–10^, like e.g. raw material composition, processing^11,12^, fiber content^13^, etc. In laboratory mice, there is a lack of data on digestibility of typical standard and experimental diets so that the exact impact of such factors cannot be estimated specifically in mice. The first step to establish more knowledge about the digestive capacity of mice is to conduct feeding trials on standard diets. This can be used to improve the feeding recommendations in a factorial calculation of energy and nutrient requirements, as established for many pet and farm animal species^8,14–16^.

In meta-analyses on different species^17–19^ and the authors´ own previous trials in mice^20^, quantitative estimations of gastrointestinal mineral absorption could be calculated from data on intake and digested amount. By regression analysis in the form of Lucas-tests^21^, it is possible to test for uniformity of absorption and estimate the true nutrient digestibility. As an example, the calcium and phosphorus homeostasis was found to differ between species groups, which can be explained by their feeding/digestive strategies^18,22^. In the present study, we aimed to apply Lucas-test to the digestibility data of crude nutrients and minerals in mice to identify species-specific patterns.

Since laboratory rodent diets differ in many aspects, we chose to start the investigation with the standard diet fed in our animal facility. The specific manufacturer or brand was not included in the aim of the trial. There are also plenty of genetic backgrounds in laboratory mice, and a potential effect of the mouse line on digestive parameters cannot be ruled out. Therefore, we used two common laboratory mouse lines, C57Bl/6 J and CD1, to test for differences in energy, crude nutrient and mineral digestibility.

## Results

### Digestibility

The voluntary daily feed intake in the CD1 mice was significantly higher than that of the C57Bl/6 J mice (36 ± 1.4 g vs. 27 ± 0.82 g per animal; *p* < 0.001). Correspondingly, the mean body weight (BW) during the trial was significantly higher in the CD1 strain (35.6 ± 0.3 g vs. 24.6 ± 0.4 g, *p* < 0.0001).

There were no significant differences between C57Bl/6 J and CD1 mice in the apparent digestibility of dry matter (DM), gross energy (GE), crude protein, crude fiber, crude fat and nitrogen-free extracts (NfE) (Table 1). Apparent digestibility of crude ash was significantly higher in the C57Bl/6 J mice (44 ± 3.6%) than the CD1 mice (34 ± 8.1%; *p* < 0.05).

**Table 1:** Apparent digestibility (%) of dry matter, gross energy, crude nutrients and minerals in C57Bl/6J and CD1 mice.

Parameter	C57Bl/6J	CD1	*p*
Dry matter	78 ± 1.1	78 ± 2.2	0.531
Gross energy	80 ± 1.0	81 ± 2.3	0.736
Crude protein	82 ± 1.1	81 ± 2.2	0.305
Crude fat	91 ± 0.94	92 ± 1.5	0.262
Crude fiber	29 ± 4.8	30 ± 6.5	0.921
Crude ash	44 ± 3.6	34 ± 8.1	< 0.05
N-free extracts	85 ± 1.0	85 ± 1.6	0.871
Calcium	27 ± 5.5	28 ± 5.5	0.627
Phosphorus	38 ± 5.5	35 ± 4.8	0.276
Magnesium	48 ± 2.9	36 ± 4.8	< 0.001
Potassium	85 ± 1.2	82 ± 2.8	< 0.05
Sodium	63 ± 5.0	70 ± 6.2	0.059

Values given in % as means ± standard deviation.

n = 6 cage units per mouse line.

The apparent digestibility of calcium and phosphorus did not differ significantly between the genetic lines (*p* = 0.362; *p* = 0.276). For magnesium (*p* < 0.001) and potassium (*p* < 0.05), the C57Bl/6 J strain had a significantly higher apparent digestibility than the CD1 stock. There was a statistical trend towards a higher apparent sodium digestibility in the CD1 mice (70 ± 6.2% vs. 63 ± 5.0%; *p* = 0.059).

### Regression of crude nutrients

The regression plots could be charted for both C57Bl/6 J and CD1 mice because of the uniform data distribution.

**Figure 1:**
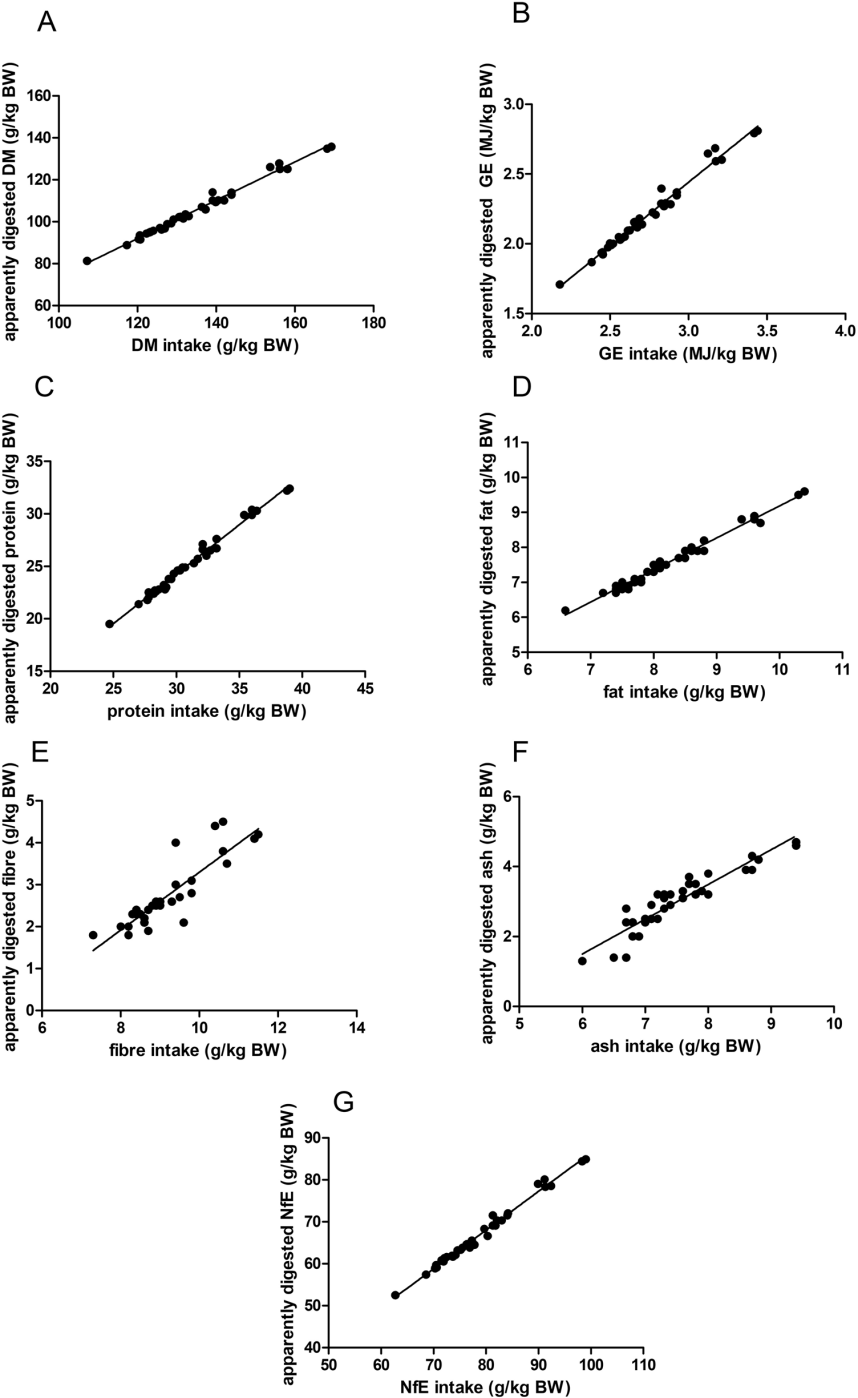
Regression plots of intake and apparently digested amount of dry matter (DM), gross energy (GE) and crude nutrients (data of C57Bl/6 J and CD1 mice). The regression equations and the parameters describing the curve fit can be found in Table 2.

There was a strong linear relationship between intake and apparently digested amount of DM (Fig. 1A), GE (Fig. 1B), protein (Fig. 1C), fat (Fig. 1D) and NfE (Fig. 1G), respectively (R^2^ > 0.95). Crude ash intake and apparently digested amount of crude ash were also correlated linearly (R^2^ = 0.86; Fig. 1F).

**Table 2:** Regression equations from crude nutrients.

	Unit	Slope	Intercept	R^2^	Sy.x
Dry matter	*g/kg BW*	0.91 ± 0.02	− 18.00 ± 2.30	0.99	1.40
Gross energy	*MJ/kg BW*	0.91 ± 0.02	− 0.38 ± 0.09	0.98	0.04
Crude protein	*g/kg BW*	0.94 ± 0.02	− 4.00 ± 0.54	0.99	0.33
Crude fat	*g/kg BW*	0.92 ± 0.02	0.02 ± 0.17	0.98	0.11
Crude fibre	*g/kg BW*	0.69 ± 0.07	− 3.60 ± 0.60	0.77	0.37
Crude ash	*g/kg BW*	0.99 ± 0.07	− 4.50 ± 0.52	0.86	0.32
N-free extracts	*g/kg BW*	0.92 ± 0.02	− 5.70 ± 1.30	0.99	0.83

On the x-axis, the intake was plotted, on the y-axis the apparently digested amount.

*BW* body weight.

The mean true digestibility calculated according to the slope of the regression equations (Table 2) was above 90% for DM (91%), GE (91%), crude protein (94%), fat (92%), ash (99%) and NfE (92%).

Mean true fibre digestibility was 69% according to the regression equation, which showed less strict linearity (R^2^ = 0.77; Sy.x = 0.37; Fig. 1E).

### Regression of minerals

**Figure 2:**
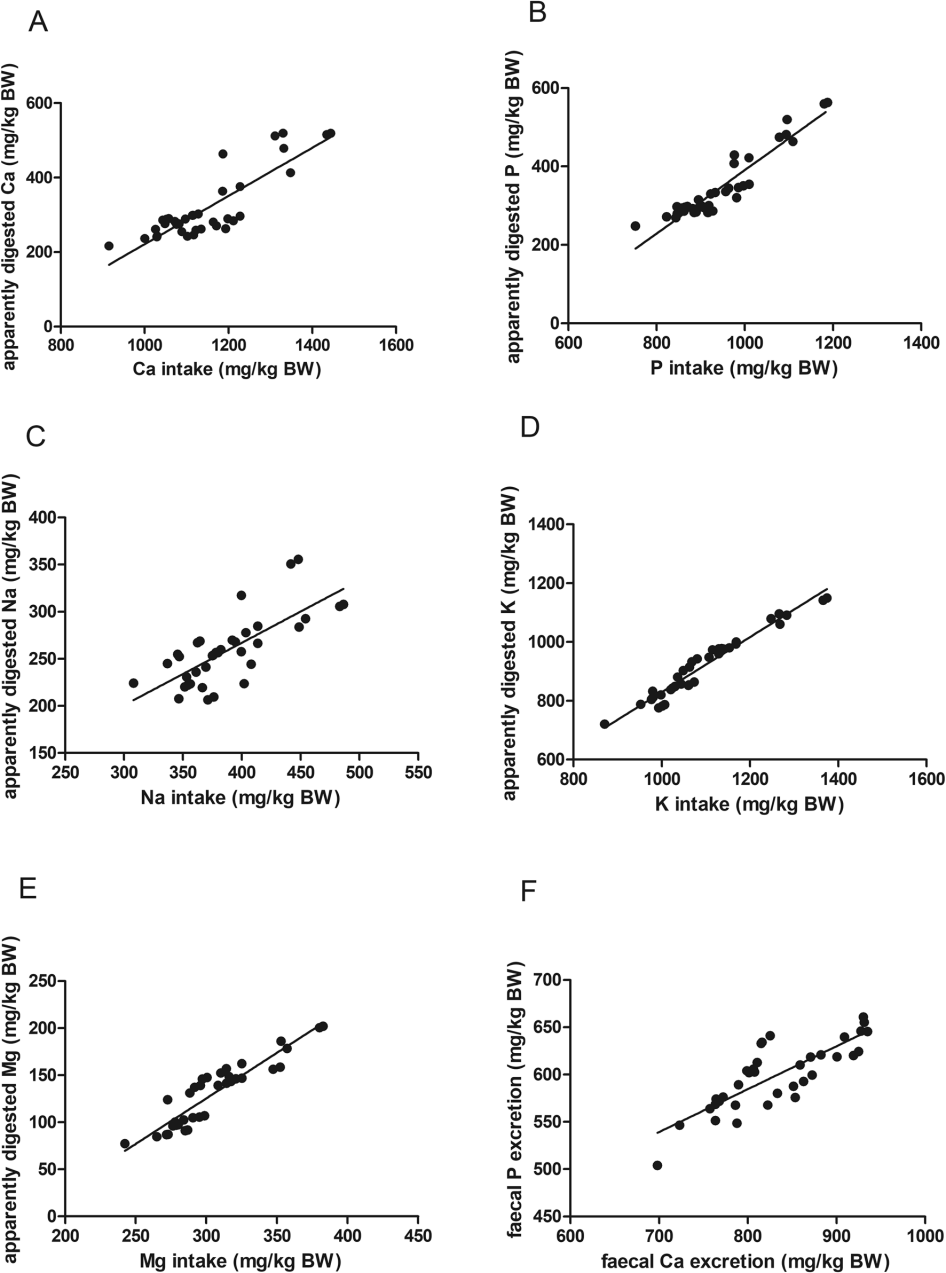
Regressions of intake and apparently digested amount of the minerals (**A**–**E**) and faecal Ca and P excretion (**F**) (data of C57Bl/6J and CD1 mice). The regression equations and parameters describing the curve fit can be found in Table 3. (*Ca* Calcium, *P* phosphorus, *Na* sodium, *K* potassium, *Mg* magnesium).

Calcium intake and apparently digested amount of calcium showed a linear correlation (R^2^ = 0.73; Sy.x = 48; Fig. 2A). The mean true calcium digestibility calculated with the equation´s slope was 65% (Table 3).

**Table 3:** Regression equations for the minerals.

	Unit	Slope	Intercept	R2	Sy.x
Calcium	*mg/kg BW*	0.65 ± 0.07	− 425 ± 77	0.73	48
Phosphorus	*mg/kg BW*	0.81 ± 0.05	− 417 ± 49	0.88	30
Magnesium	*mg/kg BW*	0.97 ± 0.07	− 165 ± 22	0.84	14
Potassium	*mg/kg BW*	0.94 ± 0.04	− 107 ± 41	0.95	26
Sodium	*mg/kg BW*	0.66 ± 0.10	1.8 ± 41	0.54	25

On the x-axis, the intake was plotted, on the y-axis the apparently digested amount.

*BW* body weight.

There was a linear correlation between phosphorus intake and the apparently digested amount of phosphorus (R^2^ = 0.88; Sy.x = 30; Fig. 2B), resulting in a mean true phosphorus digestibility of 81%.

For sodium, the linearity of the correlation was less pronounced (R^2^ = 0.54; Sy.x = 25; Fig. 2C), with a mean true sodium digestibility of 66%. If data was separated between C57Bl/6 J and CD1 mice, the fit of the regression line was better for each line (Supplementary Fig. S1). For the C57Bl/6 J strain, the regression equation was y = 0.71 ×− 32 (R^2^ = 0.71; Sy.x = 18) and for the CD1 stock y = 1.1 ×− 137 (R^2^ = 0.79; Sy.x = 19). The comparison of the regression lines rendered a p-value of 0.050 for the slope and a significant difference in the intercepts (*p* < 0.0001).

Potassium intake and apparently digested amount of potassium showed a strong linear correlation (R^2^ = 0.95; Sy.x = 26; Fig. 2D). The resulting mean true digestibility of potassium was 94%.

There was a linear correlation between magnesium intake and apparently digested magnesium (R^2^ = 0.84; Sy.x = 14; Fig. 2 E). The mean true magnesium digestibility calculated from the slope was 97%.

If faecal calcium excretion (x-axis) and faecal phosphorus excretion (y-axis; both in mg/kg BW) were correlated, the plot showed a medium strength linearity (y = 0.45 x + 222; R^2^ = 0.67; Sy.x = 21; Fig. 2F).

## Discussion

In the present study, the mice were group-housed to pool faecal samples per cage. This is a reliable method for digestibility trials in mice^11,20^ for several reasons: Mice as social animals prefer to live in groups. By pooling the faeces per cage, the time of sampling can be kept short to minimize the stress for the mic and it also reduces the inter-individual variation which adds reliability to the results.

The apparent digestibility of GE did not differ significantly between the mouse lines (*p* = 0.736), neither did protein, fat and fiber digestibility. Considering that the BW of both lines were within the usual range given by the breeder (Charles River, Germany), the differences in BW are likely related to the voluntary feed intake in the CD1 stock, not digestibility. With a mean apparent GE digestibility of about 80%, the values reported in the present study match the digestibility found in C57Bl/6 J mice in a previous trial^11^ and the range reported for rats^7,10^. If we apply the mean apparent digestibility of ~ 80.5% to the analyzed GE content of the diet (18.4 MJ GE/kg, as fed), this results in a digestible energy (DE) content of 14.8 MJ DE/kg diet. Calculating the metabolizable energy (ME) with the equation for pig mixed feed^8^ as proposed in literature^7^, the result would be 13.2 MJ ME/kg diet, about 72% of the original GE content. Future studies into energy evaluation for mice are necessary, quantifying renal energy losses.

For the energy sources crude protein, crude fat and NfE, there was also no significant difference between the C57Bl/6 J and the CD1 mice. The apparent digestibility values of crude protein and crude fat were in the same range as the values reported in literature in C57Bl/6 J mice^11^ and in rats^10^. Compared to a feeding trial in rats^23^, where apparent GE digestibility on a high fibre and a low fibre diet is reported as 82% and 93%, respectively, the values found in this study are in the same dimension as the rat high fibre diet. The rats fed a control diet in another trial^24^ had a higher apparent digestibility of DM, GE, crude protein and crude fat than the mice in this study. The crude fibre content of that diet (4.1% DM) was lower than in the diet used in the present study (6.8% DM), which may contribute to the difference. Though mice and rats differ in several aspects of digestive physiology^25,26^, the overall dimension of energy and crude nutrient utilization from standard diets in the laboratory setting seems to be comparable. A comparison of both species on the same diets under standardized conditions is needed to elucidate this aspect further.

Using the Lucas-test, the mean true digestibility of the nutrients could be estimated. The distribution of data allowed plotting both C57Bl/6 J and CD1 mice in one graph. For DM, GE, crude protein, crude fat and NfE, an extremely strong correlation between intake and apparently digested amount could be shown (R^2^ > 0.95), resulting in true digestibility values of > 90%. The standard “natural” diets (based on cereals) for laboratory rodents seem to be of high overall digestibility. Due to the extremely strong linearity, the intake of the respective nutrient seems to be the main determinant of the apparently digested amount while the digestibility coefficient remains stable.

The true digestibility of crude fibre was lower than that of the other macronutrients (69%), and the linearity observed in the Lucas-test was less pronounced (R^2^ = 0.77). This is not surprising, given that fibre cannot be digested prae-caecally by the mammalian digestive enzymes. In the murine gastrointestinal tract, fibre undergoes microbial fermentation mostly in the caecum^25^, rendering short-chain fatty acids that can be utilized as source of energy to a certain degree. The higher variability in fibre digestibility may be due to inter-individual differences in the extent or effectiveness of microbial fermentation and the microbiota composition in the gastrointestinal tract. Fibre digestibility was lower than that of guinea pigs (41%), similar to that of degus (33%) and higher than that of leaf-eared mice (18%)^13^. Setting fibre utilization in the context of other species gives a better picture of dietary specialization of laboratory mice in a comparative view.

The C57Bl/6 J mice had a significantly higher apparent digestibility of crude ash than the CD1 mice, which corresponded with a significantly higher apparent digestibility of magnesium and potassium in the C57Bl/6 J strain (Table 1). Only for sodium, the CD1 stock had a trend towards significantly higher apparent digestibility.

Apparent calcium digestibility did not differ significantly between the mouse lines and ranged round 30%, which is similar to the apparent calcium digestibility of the rats fed the control diet in a study by Frommelt et al.^24^. Mice are omnivorous hindgut fermenters with an enlarged caecum^25^, so it seems logical to compare these values to other hindgut fermenters. The observed apparent calcium digestibility in the mice of this study was considerably lower than that of other hindgut fermenters (e.g. 44–60% in horses^27–29^; 40–60% in elephants; 60–88 in rhinoceros^30^; up to 65% in degus^31^ and up to 83% in rabbits^32^, depending on intake). This difference might be due to the different natural type of diet (herbi-omnivorous vs. herbivorous), but can only be speculated upon in this study. The Lucas-test for calcium in the mice showed sufficient linearity (R^2^ = 0.73) and the resulting mean true digestibility of 65% is much higher than the value of 33% found in C57Bl/6 J mice in four diet groups in a previous study^20^. The feed intake per mouse per day was considerably lower in the present study, which may have a positive influence on calcium absorption via faecal DM excretion. In carnivores, for example, a correlation between faecal DM excretion and faecal calcium excretion is known^33,34^. The dataset of the present study also revealed a strong correlation between faecal DM and calcium excretion (mg/kg BW; y = 6.92 x − 85.3; R^2^ = 0.88), to that a similar bulking effect is likely.

The apparent phosphorus digestibility of both mouse lines in this study was slightly higher than that found previously in C57Bl/6 J strain^20^. Phosphorus intake and apparently digested amount of phosphorus were correlated linearly (R^2^ = 0.88) and the mean true phosphorus digestibility amounted to 81%. The slope of the linear correlation between the faecal excretion of calcium and phosphorus (0.45; Fig. 2F) translates to faecal calcium excretion that is roughly twice the faecal phosphorus excretion. This is similar to the finding in carnivores^18^, where the combined excretion of calcium and phosphorus in the form of insoluble complexes is suspected. Under the maintenance supply with both minerals, the apparently digested amount seems to be determined predominantly by the intake of the respective nutrient (Lucas-tests, Fig. 2A,B) without obvious regulation of intestinal absorption. The linear correlation of the faecal excretion of calcium and phosphorus points to a chemical effect of solubility / complex formation. It needs to be investigated in further experiments with an intake of each mineral at marginal and below-requirement levels whether and how the regression in the Lucas-test changes. For example during high calcium supply, renal excretion may play a role in calcium excretion as reported for wild wood mice (*Apodemus sylvaticus*)^35^. In times of low calcium supply, intestinal absorption may be increased^36^, which would alter the regression plot^17^, or stay unchanged, which would point to skeletal regulation of calcium homeostasis^18^.

The C57Bl/6 J mice hat a significantly higher apparent magnesium digestibility than the CD1 mice (*p* < 0.001) and C57Bl/6 J mice in literature^20^. The Lucas-test showed a strong linear correlation between magnesium intake and the apparently digested amount over data from both genetic lines, resulting in a nearly complete true digestibility (97%). Magnesium homeostasis is regulated by the renal excretion/reabsorption to a large extent in mice and rats^37,38^, while potential changes in apparent digestibility during low or high magnesium intake in rodents have not been studied to the authors´ knowledge.

Both genetic lines had a high (> 80%) apparent potassium digestibility, with significantly higher values in the C57Bl/6 J mice. As expected, the correlation between potassium intake and apparently digested amount was extremely strong (R^2^ = 0.95). A mean true digestibility of 97% could be calculated from the slope, indicating a nearly total absorption of the ingested amount. This corresponds with the reports of a fast absorption of potassium in the intestinal tract, majorly the colon as site of active potassium absorption and secretion^39,40^. The high digestibility seems to be necessary to maintain adequate potassium levels in the body, because there are no mechanisms conserving potassium^39^.

Sodium intake and apparently digested amount of sodium of both mouse lines together showed a weaker linearity than the other nutrients (R^2^ = 0.66). The fit of the regression lines improved when separated into C57Bl/6 J and CD1 (R^2^ = 0.71 and 0.79, respectively; Supplementary Fig. S1). There seems to be a statistical trend towards a higher true sodium digestibility in CD1 mice, as indicated by the comparison of the regression lines. This is in accordance with the near-significant higher apparent sodium digestibility in the CD1 mice (70% vs. 63% in C57Bl/6 J; *p* = 0.059) and the higher urine pH in CD1 mice found previously^41^. Sodium is positively charged and acts as an alkalizing compound in the equation of the dietary cation–anion-balance^8,42–44^. The effect of sodium on acid–base-balance may depend on the sodium compound that is the major source in the diet and the relative absorption of the other ions of the compound. For example in horses, sodium chloride has an acidifying effect independent of the dietary cation anion balance that may be linked to the relative absorption of all minerals and an effect on the intermediate mineral metabolism^27^. Further research is warranted to gain more insights into the mechanisms behind this, e.g. renal mineral excretion.

Solely from digestibility data, it is impossible to explain where the differences in apparent digestibility of magnesium, potassium and sodium between C57Bl/6J and CD1 mice observed in this study come from. It seems likely that the intestinal absorption might somehow differ. This has to be investigated in studies with other methods, e.g. Ussing chamber to measure flux rates or assessing the intestinal transporters on a molecular level. On the organism level, differences in mineral absorption can influence e.g. the dietary requirement, the mineral homeostasis and the acid base balance.

In the reported study, we used male mice. In animal nutrition, sex effects on nutrient digestibility under maintenance conditions (i.e. not in gestation or lactation) are not known from other species, so an effect of sex is not expected to play a role in this study. Energy and nutrient absorption during growth and reproduction needs to be studied in the future because this can be expected to differ markedly from maintenance data. Digestibility and requirement is known to differ between growth and adulthood, and it might also be worth investigating old age for potential differences. In this study, we report data for young adult age under maintenance conditions.

In conclusion, the data reported in this study is valuable as reference data for diet digestibility in C57Bl/6 J and CD1 mice under standard conditions, on a pelleted rodent diet. The commonly used mouse lines do not differ in apparent digestibility of energy and crude nutrients, but in the case of some minerals. Further feeding trials are needed to find out whether low or high supply of single nutrients change the digestibility coefficient, indicating regulation of intestinal absorption. For future trials, diets from several manufacturers with different composition, processing and, most important, nutrient contents, need to be used. In this way, potential influencing dietary factors on nutrient digestibility or nutrient interactions can be identified. If the difference in mineral digestibility between mouse lines wan be confirmed in further trials, the dietary mineral levels might even need to be adapted to the lines. This knowledge contributes towards the *Refinement* of laboratory mouse feeding and planning of experiments, as well as to the interpretation of results.

## Methods

The trial was approved by the ethical committee of the Veterinary Faculty, LMU München (reference no. 264-13-04-2021). Housing of laboratory mice was in accordance with European and German animal welfare legislations (5.1-231 5682/LMU/BMC/CAM 2019-0007). Room temperature ranged from 20 to 22 °C, relative from 45 to 55%. The light cycle was adjusted to 12 h light:12 h dark period. Room air was exchanged 11 times per hour and filtered with HEPA-systems. Hygiene monitoring was performed every three months based on the recommendations of the FELASA-14 working group.

### Animals

Male mice of two commonly used laboratory mouse lines were used starting at 8 weeks of age (n = 18 each): C57Bl/6 J inbred mice (Charles River, Germany) and CD1 outbred mice (Crl:CD1(ICR), Charles River, Germany). Breeding and rearing of the mice was done in the same facility as the trial under standardized conditions. The mice were housed under specified-pathogen-free conditions in individually ventilated cages (Type II long;Tecniplast, Buggugiate, Italy) in groups of three on silicate bedding (Tigerino Crystals, Matina GmbH, Munich, Germany). The cages were equipped with nesting material (5 × 5 cm Nestlet, Datesand, UK) and a red corner house (Tecniplast). The mice were weighed weekly throughout the trial and a mean body weight (BW) for the trial period was calculated per animal. The mice were not sacrificed for this study and no chemicals were administered.

### Diets

The standard diet for rodents used in the facility was fed during the trial (Altromin 1314P, irradiated at 25 kGy, 10 mm pellets; Altromin Spezialfutter GmbH, Lage, Germany). It was available ad libitum, and water (UV-treated, filtered, partially desalinated) was accessible at all times. Table 4 gives the analyzed nutrient content of the batch used in the trial.

**Table 4:** Analyzed composition of the diet used in the trial (as fed basis unless stated otherwise).

Nutrient	Content in the diet
Gross energy (MJ/kg)	18.4
Dry matter (%)	89.8
Crude protein (%)	20.7
Crude fat (%)	5.5
Crude fibre (%)	6.1
Crude ash (%)	5.0
N-free extracts (%)	52.6
Calcium (%)	0.77
Phosphorus (%)	0.63
Magnesium (%)	0.20
Potassium (%)	0.73
Sodium (%)	0.26

### Feeding trial

Because the diet was the standard diet in the facility, no specific adaptation period was necessary. Over a period of 7 days, the daily feed intake was recorded by weighing the offered and the leftover amount of feed. In addition, the total amount of faeces per cage was collected every day, weighed and stored at − 20 °C until further analyses. Feed intake and faecal collection was performed once per day, roughly around the same time, to account for the last 24 h.

### Analyses

A representative sample of the diet and the complete faecal samples (pooled per cage over 7 days) were homogenized and analyzed for gross energy (GE; bomb calorimetry), crude nutrients (Weende analysis; VD LUFA 2012) and minerals (calcium, sodium, potassium: flame emission spectrometry [EFOX 5053, Eppendorf AG, Hamburg, Germany]; magnesium: atom spectroscopy [Analyst 800, no. 90454 040301, Perkin Elmer, Waltham, MA, USA]; phosphorus: photometry [GENESYS 10 UV, Thermo Spectronic, Rochester, NY, USA]). The nitrogen-free extracts (NfE) were calculated by subtraction of crude protein, crude fat, crude ash and crude fiber from the dry matter (DM) content.

### Calculations and statistics

For each nutrient, the apparent digestibility was calculated as follows:

$$apparent \,digestibility \left(\%\right)=\frac{\left(intake\, of \,nutrient-faecally \,excreted \,amount \,of \,nutrient\right)}{intake\, of \,nutrient}x 100$$

The two mouse lines C57Bl/6 J and CD1 were compared in terms of BW, diet intake and apparent digestibility via two-tailed, unpaired Mann–Whitney test (no normal distribution assumed). The level of significance was defined as α = 0.05 (GraphPad Prism^®^ 5.04, Graphpad Software, San Diego, CA, USA).

Lucas-test regression plots were created for DM, GE and each nutrient^21^. The nutrient intake is plotted on the x-axis, the apparently digested amount of the nutrient on the y-axis (both in mg/kg BW) so that the slope of the regression line, multiplied by 100, gives the mean true digestibility of the nutrient. The strength of the linear regression was expressed with the correlation coefficient R^2^ and the standard error of the regression Sy.x. In the case of sodium, data distribution of C57Bl/6 J and CD1 mice was not uniform, so that separate regression plots were created. The test of Ho was used to compare the resulting regression equations in terms of statistical significance (BiAS. For Windows).

### Statement on ARRIVE guidelines

Hereby we confirm that the study is reported in accordance with ARRIVE guidelines for animal studies.

### Supplementary Information

Supplementary Figure S1.

## Data Availability

The datasets generated during and/or analysed during the current study are available at biostudies with the accession number S-BSST1228 (https://www.ebi.ac.uk/biostudies/studies/S-BSST1228).
