# Supplementary material for: Digestibility of crude nutrients and minerals in C57Bl/6J and CD1 mice fed a pelleted lab rodent diet
Source: Sci Rep. 2024 Jan 20;14:1791. doi: 10.1038/s41598-024-52271-5 (PMC10799863; doi:10.1038/s41598-024-52271-5)
Supplement: Supplementary file 1 — Supplementary Figure S1. [file 41598_2024_52271_MOESM1_ESM.pdf]

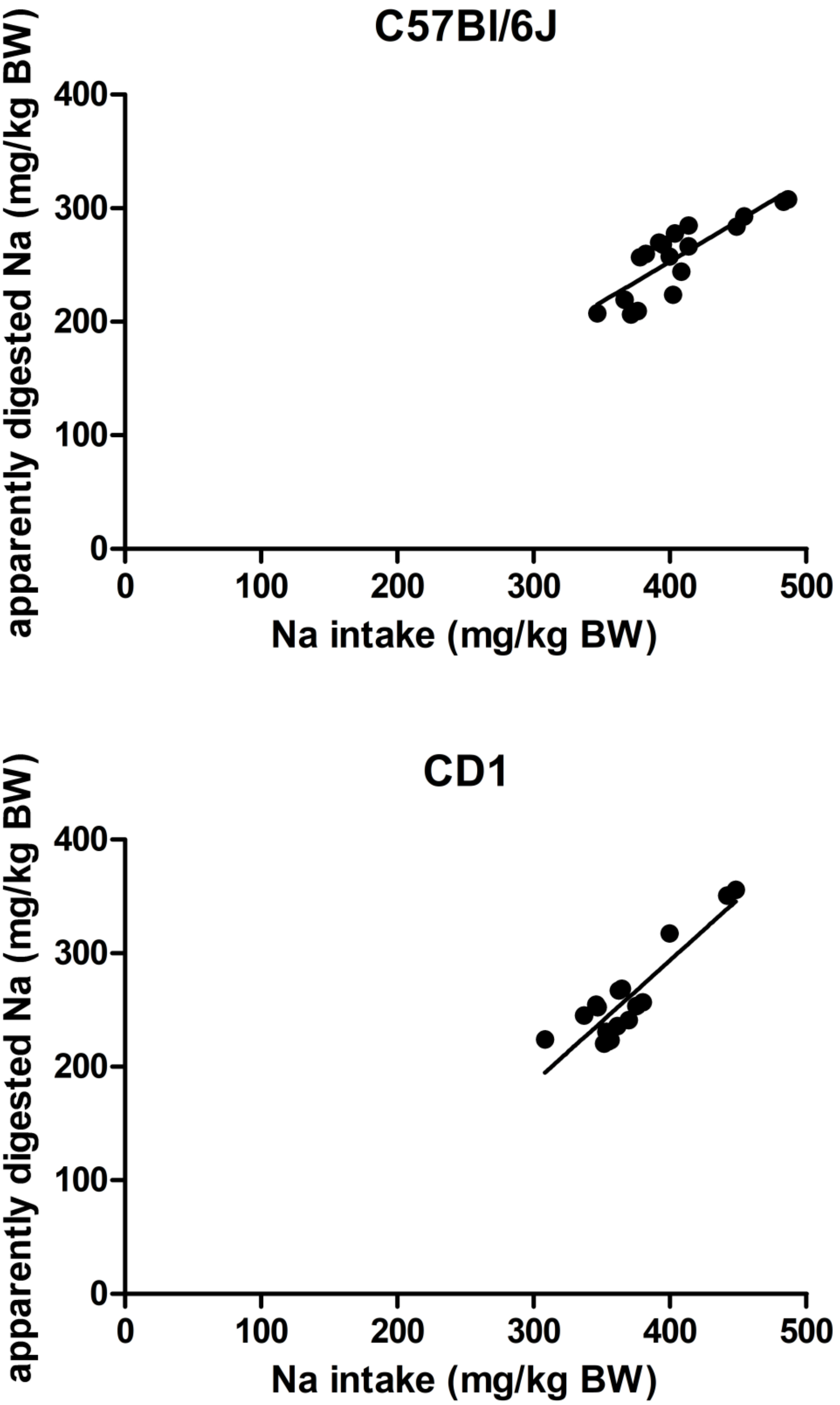

Supplementary figure S1. Regression plots of sodium intake and apparently digested amount of sodium with data separated according to mouse line.
